# P2Y12 receptor as a new target for electroacupuncture relieving comorbidity of visceral pain and depression of inflammatory bowel disease

**DOI:** 10.1186/s13020-021-00553-9

**Published:** 2021-12-20

**Authors:** Yanzhen Li, Hong Zhang, Jingwen Yang, Muouyang Zhan, Xuefei Hu, Yongmin Liu, Lingling Yu, Xiaochen Yan, Shangdong Liang, Ruyue Zhang, Ying Lu, Beining Li, Cunzhi Liu, Man Li

**Affiliations:** 1 Department of Neurobiology, School of Basic Medicine, The Institute of Brain Research, Tongji Medical College of Huazhong University of Science and Technology, Wuhan, China; 2 International Acupuncture and Moxibustion Innovation Institute, School of Acupuncture-Moxibustion and Tuina, Beijing University of Chinese Medicine, Beijing, 100029 China; 3 Institute of Integrated Traditional Chinese and Western Medicine, Tongji Hospital, Tongji Medical College, Huazhong University of Science and Technology, Wuhan, 430030 China; 4 Department of Physiology, Basic Medical College of Nanchang University, Nanchang, 330006 China; 5 Xiangya Hospital of Central South University, Changsha, 410000 China

**Keywords:** P2Y12 receptor, Visceral pain, Depression, EA, IBD

## Abstract

**Background:**

The P2Y12 receptor is a kind of purinoceptor that is engaged in platelet aggregation, and P2Y12 inhibitors have been used in clinical antithrombotic therapy. The P2Y12 receptor in microglia induces interleukin-1β (IL-1β) expression, which is a key mediator of depression in the brain. Although peripheral P2Y12 is involved in neuropathic pain, whether P2Y12 expression in the medial prefrontal cortex (mPFC) is associated with comorbidities of visceral pain and depression remains unclear. Accumulating evidence suggests that electroacupuncture (EA) is effective in treating inflammatory bowel disease (IBD), but its mechanism is unknown. This study aimed to determine whether P2Y12 expression in the mPFC is associated with comorbidities of visceral pain and depression in IBD and whether EA treats IBD by targeting the P2Y12 receptor.

**Methods:**

We used 2,4,6-trinitrobenzene sulfonic acid (TNBS)-induced IBD mice. P2Y12 short hairpin RNA (shRNA) was stereotaxically injected into the bilateral mPFC. EA was performed on bilateral “Dachangshu” (BL25) acupoints once a day for 7 days. Von Frey filaments and colorectal distension were used to detect the mechanical pain threshold and visceral pain sensitivity. The sucrose preference test, tail suspension test and forced swimming test were used to evaluate depression in mice. Western blotting was used to test the expression of P2Y12 and IL-1β. Immunofluorescence staining was used to assess microglial activity.

**Results:**

We found that IBD mice presented visceral pain and depression associated with increased P2Y12 expression in the mPFC. P2Y12 shRNA significantly attenuated visceral pain and depression in IBD mice. P2Y12 shRNA significantly downregulated IL-1β expression and inhibited the activation of microglia in the mPFC of IBD mice. Meanwhile, EA played a similar role of P2Y12 shRNA. EA significantly downregulated P2Y12 expression, weakened the activation of microglia, and then inhibited IL-1β expression in the mPFC, thus relieving visceral pain and depression in IBD mice.

**Conclusion:**

The present study provided new ideas that the P2Y12 receptor in the mPFC could be a new target for the treatment of comorbid visceral pain and depression by EA. This may not only deepen our understanding of the analgesic and antidepressant mechanisms of EA but also promote the application of EA to treat IBD.

**Supplementary Information:**

The online version contains supplementary material available at 10.1186/s13020-021-00553-9.

## Background

Comorbidity of visceral pain and depression occurs with extremely high prevalence at any age [1]. A growing body of evidence has shown that patients with inflammatory bowel disease (IBD) have a higher incidence of comorbidities of depression and anxiety than healthy persons [2, 3]. Similarly, depression is associated with an increased risk of incident IBD [4, 5]. Although new therapeutic strategies in IBD have transformed from symptom control to treat-to-target algorithms, emotional symptoms are often ignored [6].

The P2Y12 receptor is a kind of metabotropic purinoceptor. It is mainly expressed in platelets, and P2Y12 inhibitors have been widely used in clinical antithrombotic therapy [7, 8]. Recently, the P2Y12 receptor was also found to be expressed in microglia, such as satellite glial cells and microglial cells [9, 10]. Inhibition of microglial P2Y12 suppressed interleukin-1β (IL-1β) release, and P2Y12-deficient microglia displayed reduced IL-1β mRNA expression and release in vitro [11]. In addition, IL-1β in the brain is a key mediator of depression-like behavior induced by acute and chronic stress [12]. Although peripheral P2Y12 is involved in the pathologic changes of neuropathic pain, whether the expression of P2Y12 in the brain is associated with comorbidity of visceral pain and depression in IBD mice remains unclear [13]. In addition, the medial prefrontal cortex (mPFC) handles the emotional and cognitive components of pain, which contributes to the development of chronic pain [14]. There is also evidence that dysfunction of the mPFC in brain activity is closely related to neuropsychiatric diseases, such as depression [15]. It is worth exploring whether the P2Y12/IL-1β pathway in microglia of the mPFC contributes to the comorbidity of visceral pain and depression in IBD.

Electroacupuncture (EA) can carry electric current to acupuncture points through needles connected to an electrical stimulator, which is transmitted to the spinal cord through sensory nerves, then to the brainstem, hypothalamus and higher center [16]. Growing evidence has shown that EA can relieve pain and induce lasting analgesia [17–19]. Studies have also revealed that EA has an antidepressant effect [20–24]. Recently, a clinical study showed that acupuncture can be used as a new therapy for the improvement of gastrointestinal symptoms and noncolonic situations, such as depression of irritable bowel syndrome [25]. However, whether EA improves the comorbidity of visceral pain and depression in IBD mice by regulating P2Y12 expression in the mPFC remains unknown, which severely hinders the clinical practice of EA in treating IBD.

Therefore, in the present study, we first investigated the change in P2Y12 in the mPFC of 2,4,6-trinitrobenzene sulfonic acid (TNBS)-induced IBD mice. To understand the role of the P2Y12 receptor in the comorbidity of visceral pain and depression, we used P2Y12 short hairpin (shRNA) to interfere with the expression of P2Y12 in the mPFC of IBD mice and then assessed visceral pain and depression-like behavior changes, as well as the protein expression of IL-1β and the activity state of microglia in the mPFC of IBD mice. Finally, we determined whether EA at bilateral Dachangshu (BL25) regulates the expression of P2Y12 in the mPFC and then inhibits the activation of microglia and the expression of IL-1β in the mPFC, thereby alleviating visceral pain and depression in IBD mice. We aimed to explore whether the upregulation of P2Y12 in the mPFC is associated with comorbidities of visceral pain and depression in IBD mice and clarify the mechanism by which EA improves the comorbidity of visceral pain and depression in IBD mice by downregulating P2Y12 expression in the mPFC.

## Materials and methods

### Animals

A total of 80 eight-week-old male C57BL/6 mice (weighing 20–25 g) were used in this study. All animals were obtained from Beijing Vital River Laboratory Animal Technology Co., Ltd. Half of the mice were randomly divided into four groups (control, TNBS, EA, and sham EA). The other mice were randomly divided into four groups (control + vectors, TNBS + vectors, control + P2Y12 shRNA, TNBS + P2Y12 shRNA). All animals were housed in cages with a 12 h light/dark cycle and were given free access to food and water. Mice were housed under a controlled environment at a constant temperature of 22–25 °C and humidity of 50 ± 10%.

### P2Y12 shRNA treatment

We amplified the shRNA coding sequence of P2Y12 by reverse transcription polymerase chain reaction and ligated it into the GV493 plasmid to generate LV-P2ry12-RNAi (Shanghai Genechem Co., Shanghai, China), and LV-GFP was used as a control. The titer of the lentivirus was 1 × 10^9^ titer units (Tu)/ml. ShRNA injections were performed three weeks before modeling. Mice were anesthetized with 10 ml/kg intraperitoneal injection of pentobarbital and fixed on a stereotaxic apparatus (RWD Instruments, China). After disinfection, the bregma and lambda points were exposed, and the balance was corrected. The coordinates were set according to the mouse brain atlas, and holes were drilled on the skull with a dental drill. Desired viral vectors were injected into the bilateral mPFC (AP: + 2.0 mm, ML: ± 0.4 mm, DV: -2.4 mm) using a microsyringe (1 μl, Hamilton, NV, USA) with a glass electrode attached to the front end. Injection occurred at a speed of 30 nl/min. P2Y12 shRNA sequence: GGTCTAGTTTGGCACGAAA and empty virus sequence: TTCTCCGAACGTGTCACGT.

### TNBS-induced IBD mice

The day before modeling, the mice were kept in a fasting and water-only environment. The mice were anesthetized with 80 mg/kg pentobarbital (20% ethanol) administered intraperitoneally. TNBS was obtained from Sigma–Aldrich, St. Louis, MO, USA. A PVC-Fr4 catheter (Φ2.7 mm, YN Medical Instrument, Yangzhou, China) was inserted at a depth of approximately 4 cm into the anus of the colon, and the other end was connected to a 1 ml syringe. Mice in the TNBS group were injected with 50 μl of TNBS (5% w/v) and 50 μl of absolute ethanol through a catheter to induce IBD [26]. Mice in the control group were injected with 50 μl of absolute ethanol and 50 μl of distilled water. After the lysate was infused, the mice were placed head down for 1 min.

### EA treatment

One day after the IBD mice were established, the mice in the EA group were treated with EA. A Han's Acupoint Nerve Stimulator (Hans-200A, Jisheng Medical Technology Co., Ltd., Nanjing, China) was used to stimulate the bilateral "Dachangshu" (BL25) of mice once a day for 7 days. EA was carried out at 1 mA and 2 Hz for 30 min. For the sham EA group, needles were inserted at the same acupoints, but Han's acupoint nerve stimulator was not connected, and no other operations were performed. During EA treatment, the mice were placed into a homemade 10 cm square denim pocket. In the control group and the TNBS group, the mice were put into the pocket without any treatment. During treatment, the mice remained awake and showed no obvious signs of distress.

### Mechanical sensitivity test

Mechanical pain was tested with von Frey filaments. Before the test, the mice were placed into the testing environment to adapt for 30 min. Before modeling with TNBS, the mice were tested for mechanical threshold for three days, and the average value was used as the baseline threshold. After the injection of TNBS, the mice were subjected to a nociceptive threshold test after EA treatment for 7 consecutive days. The mechanical threshold was measured by the “up and down” method. The mouse was placed in an organic plastic box with a metal mesh pad at the bottom, and von Frey filaments were used to apply a vertical force to the mouse's left hind paw for 6 s. Lifting the foot or licking was considered a positive response, and the statistics were calculated as the average of two tests performed at a 5-min interval.

### Visceral hyperalgesia test

After other behavioral experiments, colorectal dilatation (CRD) was used to evaluate the degree of visceral hyperalgesia, and the abdominal withdrawal reflex (AWR) of the mice was recorded. The scoring standard refers to Al-Chaer's method [27]: no behavioral response to CRD, scored as 0 points; given to stimulated mice with short head or body movements, scored as 1 point; abdominal muscle contraction during stimulation, scored as 2 points; lifted abdomen, scored as 3 points; body arched and the pelvis and scrotum are lifted, scored as 4 points. Before the test, the mice were placed in a plexiglass compartment (20 cm × 20 cm × 10 cm) for 5 min. A children's scalp needle infusion set was used as a catheter. One end of the catheter was connected to the balloon, and the other end was connected to a 10 ml syringe and a blood pressure meter through a three-way joint. The balloon was inserted into the rectum until the catheter was positioned into the anus (2 cm away from the end of the balloon). The catheter was fixed at the bottom of the tail to prevent it from falling off, and the CRD test was performed with stepwise pressure (20/40/60/80 mmHg). Each pressure value was measured twice, lasting 30 s, with an interval of 4 min, and the average value was taken.

### Sucrose preference test

Mice were single housed in cages with a water shortage for 6 h and were given two identical water bottles for 24 h, one containing sucrose water (1.5%) and the other containing ordinary water. Twelve hours later, the positions of the two bottles were changed. The weight of the water bottle was recorded before and after the test. A mouse’s preference for sucrose was recorded as sucrose consumption (g)/ (sucrose consumption (g) + water consumption (g)).

### Forced swimming test

Mice were placed in a plastic cylinder filled with water to 20 cm from the bottom under usual illumination. The behavior of mice was recorded by a camera. The duration of immobility was counted by the same person during the study as the time when the animals remained floating without any movement of either the head or the four limbs. Only the last 4 min of trials were analyzed.

### Tail suspension test

A tape was attached from the tip to a 1–2 cm position on the tail of the mouse, and the mouse was hung at a height of 30 cm on a horizontal rod. A cylindrical plastic tube was placed at the bottom of the tail to prevent the tail from climbing. The mice were suspended for 6 min, and video recordings of quantitative tests were obtained. The total time spent in the stationary posture was measured.

### Enzyme-linked immunosorbent assay

IL-1β levels were measured by a Mouse Interleukin 1β (IL-1β) ELISA Kit (Chuangxiang Biological Technology Co., Shanghai, China) as described in the manufacturer’s instructions. The tissues were collected using 0.09% NaCl and then centrifuged for 30 min at 3000 g at 4 °C within 30 min of collection. Ten microliters of testing sample and 40 µl of sample diluent were added to the testing sample well. Then, 50 µl of standard was added to the standard well, and the blank well did not contain anything. Next, 100 µl of HRP-conjugated reagent was added to each well, covered with an adhesive strip and incubated for 60 min at 37 °C. Chromogen solution A (50 µl) and chromogen solution B (50 µl) were added to each well and incubated for 15 min at 37 °C in the dark, and then stop solution was added to each well. A microtiter plate reader was used to read the optical density (O.D.) at 450 nm. The IL-1β concentration in the brain tissues was calculated from standard curves.

### Quantitative real-time polymerase chain reaction

Mice were anesthetized, and the mPFC was isolated immediately. Total RNA samples were extracted using TRIzol Reagent (Takara, Shiga, Japan). The PrimeScript PT reagent kit (Vazyme, Nanjing, China) was used to synthesize cDNA. Quantitative real-time PCRs were performed with the SYBR Green Kit (Vazyme, Nanjing, China) using 1 μl of cDNA and 0.2 μl of each primer in a 10 μl final volume. Primers were designed through the National Center for Biotechnology Information using the following sequences: P2Y12, forward primer TCACCCAGGTTCTCTTCCCA, reverse primer CGGCTCCCAGTTTAGCATCA; β-actin, forward primer TGCTGTCCCTGTATGCCTCTG, reverse primer TGATGTCACGCACGATTTCC. The comparative Ct method was used for data analysis.

### Western blot

The protein of the collected mouse brain tissues was extracted using cold lysis buffer containing protease inhibitor. The protein concentrations were determined by the bicinchoninic acid (BCA) method (Beyotime Biotechnology, Shanghai, China). Equal amounts of protein from different samples were separated using sodium dodecyl sulfate–polyacrylamide gel electrophoresis (SDS–PAGE) gels and then electrotransferred onto a 0.45-μm polyvinylidene fluoride (PVDF) membrane (Millipore, USA). After the membrane was immersed in 3% bovine serum albumin (BSA) for 2 h, target proteins were detected using primary antibodies, including rabbit anti-P2Y12R antibody (1:800), rabbit anti-IL-1β antibody (1:800), and rabbit anti-β-actin antibody (1:1000). Horseradish peroxidase (HRP)-conjugated secondary antibodies were incubated at 37 °C for 40 min. Immunoreactive bands on the membrane were visualized by enhanced chemiluminescent detection (Beyotime Biotechnology, Shanghai, China). The relative expression abundance was tested by ImageJ software (NIH, Bethesda, MD, USA).

### Immunofluorescence

Animals were anesthetized after the behavioral experiment. The brains were dehydrated for 24 h in 10% sucrose, 20% sucrose and 30% sucrose in succession after postfixation in 4% paraformaldehyde overnight. These samples were cut into 20 μm thick coronal sections and then used for rabbit anti-IBA-1(1:200) or mouse anti-CD68 (1:400) immunohistochemistry staining. Then, the tissues were incubated with secondary antibodies from Jackson ImmunoResearch: donkey anti-rabbit IgG conjugated with donkey anti-rabbit IgG conjugated with DyLight 594 (1:600), and donkey anti-mouse IgG conjugated with donkey anti-mouse IgG conjugated with DyLight 594 (1:400). The reaction product was visualized and captured by a digital fluorescence microscope (Olympus), the percentage of IBA-1-positive cells was quantified by Strata Quest 7.0 and the percentage of CD68-positive area was quantified by ImageJ software.

### Statistical analysis

All data are presented as the mean ± SEM. GraphPad Prism 8 software (Inc., La Jolla, CA, USA) was used in this study for data analysis. A t test was used to analyze significant differences between two groups. Ordinary one-way analysis was used, followed by Tukey’s multiple comparisons test. For visceral pain and mechanical sensitivity, two-way multiple comparisons with Bonferroni’s multiple comparisons test were used. A value of *P* < 0.05 was considered statistically significant.

## Results

### TNBS-induced IBD mice presented comorbidities of visceral pain and depression and upregulation of P2Y12 expression in the mPFC of IBD mice

**Fig. 1:**
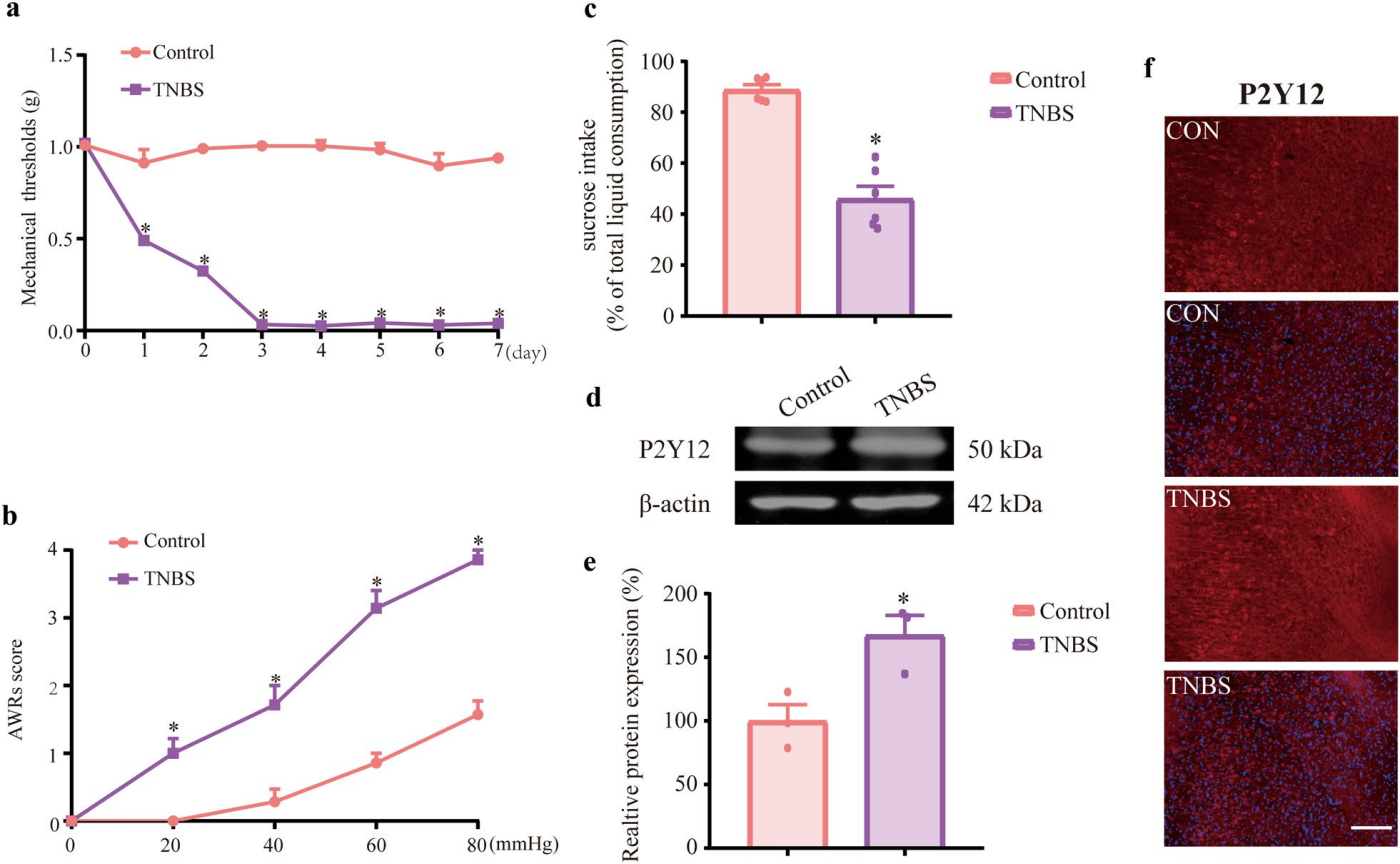
TNBS-induced IBD mice presented comorbidities of visceral pain and depression and P2Y12 expression in the mPFC was upregulated in IBD mice. **a** Time course of mechanical threshold in response to von Frey filaments (n = 6–8 mice). **b** Visceral hyperalgesia was evaluated by colorectal dilatation (CRD) (n = 6–8 mice). **c** Sucrose preference test (SPT) was used to evaluated depression-like behaviors. Mice’s preference for sucrose was recorded as: sucrose consumption(g) / (sucrose consumption(g) + water consumption(g)) × 100% (n = 6–8 mice). **d** Representative immunoblots of P2Y12 and β-actin protein expression in mPFC. **e** Densitometric analysis of P2Y12 protein normalized to the loading control (n = 3 mice). **f** P2Y12 are stained red and nuclei are stained blue with DAPI in the mPFC of IBD mice. Scale bars: 100 μm. The data are expressed as mean ± SEM. *p < 0.05, compared with the control group

Von Frey filaments were used to evaluate mechanical allodynia. The mechanical pain threshold of mice was remarkably reduced in the TNBS group compared with that of the control group (Fig. 1a,  P < 0.05). The AWR score of CRD was used to assess visceral hypersensitivity in IBD mice. The AWR score in the TNBS group was higher than that of the control group (Fig. 1b, P < 0.05). SPT was used to evaluate depression-like behavior in IBD mice. The intake of sucrose water in the TNBS group was lower than that of the control group (Fig. 1c,  P < 0.05). We tested the expression of P2Y12 in IBD mice by western blotting, and P2Y12 expression was upregulated in the TNBS group compared with the control group (Fig. 1d, e,  P < 0.05). Fluorescence immunohistochemistry showed similar results (Fig. 1f). In summary, mechanical allodynia, visceral hypersensitivity and depression-like behaviors occurred, and the expression of P2Y12 was upregulated in the mPFC of IBD mice.

### P2Y12 shRNA treatment relieved the comorbidity of visceral pain and depression in IBD mice

**Fig. 2:**
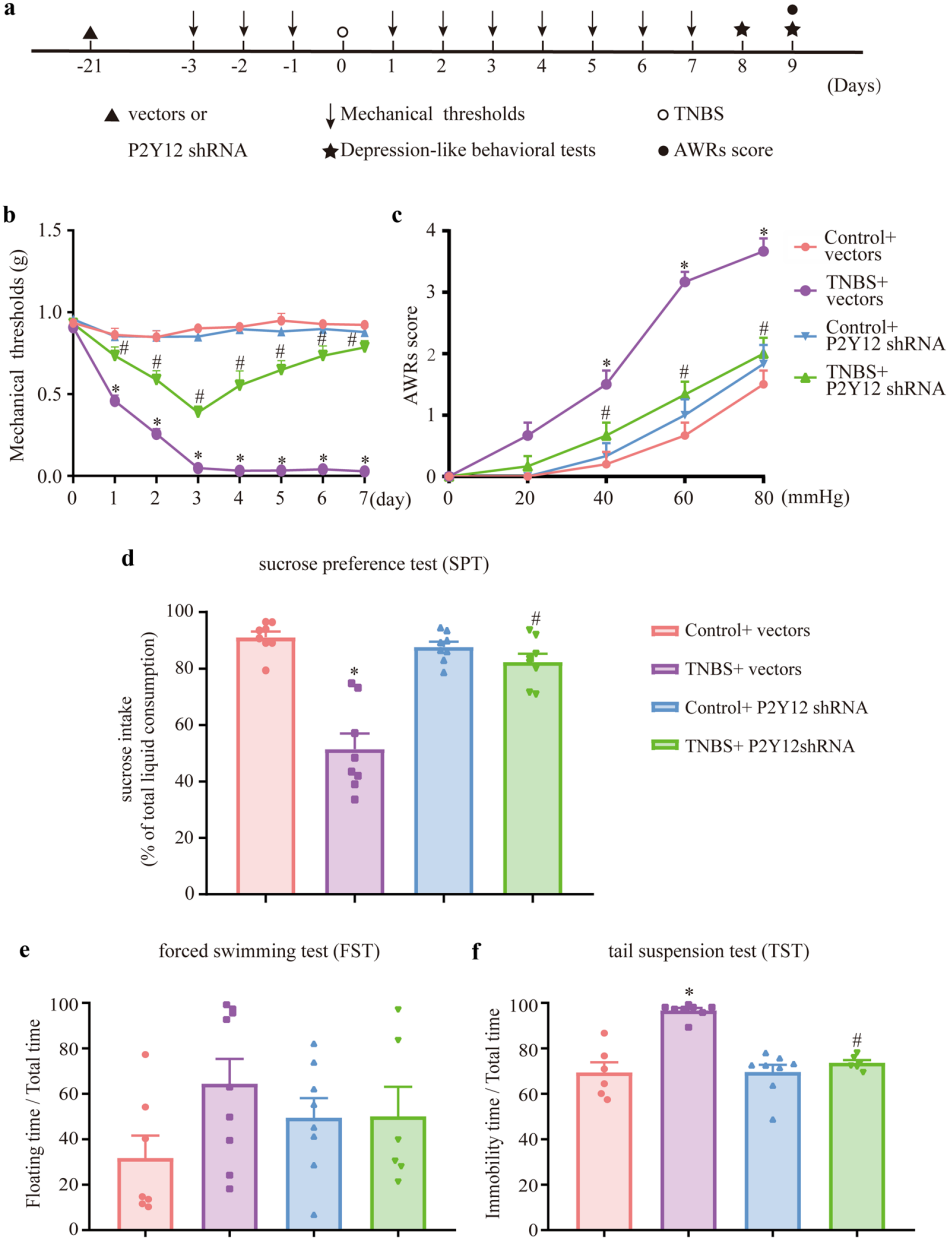
P2Y12 shRNA relieved the mechanical allodynia, visceral hyperalgesia and depression-like behaviors in IBD mice. **a** Experimental flowchart. **b** Time course of mechanical threshold with P2Y12 shRNA treatment. **c** Visceral hyperalgesia was evaluated by CRD. **d** After P2Y12 shRNA treatment, mice’s preference for sucrose was recorded as: sucrose consumption(g) / (sucrose consumption(g) + water consumption(g)) × 100%. **e** Representative results of the effect of the P2Y12 shRNA treatment on the proportion of floating time in the FST. The proportion of floating time was recorded as: (Floating time(s)/total time(s)) × 100%. **f** Representative results of the effect of the P2Y12 shRNA treatment on the proportion of immobility time in the TST. The proportion of immobility time was recorded as: (Immobility time(s)/total time(g)) × 100%. The data are expressed as mean ± SEM (n = 6–9 mice). *p < 0.05, compared with the control + vectors group; #*p* < 0.05, compared with the TNBS + vectors group

To demonstrate whether P2Y12 shRNA treatment alleviated mechanical allodynia and visceral pain in IBD mice, von Frey filaments and the AWR score of CRD were used. A schematic diagram of P2Y12 shRNA injection (Additional file 1: Fig. S1a, b) and RT–PCR was used to analyze transfection efficiency (Additional file 1: Fig. S1, c). Compared with the control + vectors group, the mechanical pain threshold was decreased in the TNBS + vectors group. Compared with the TNBS + vectors group, the mechanical pain threshold was remarkably increased in the TNBS + P2Y12 shRNA group (Fig. 2b,  P < 0.05), which revealed that P2Y12 shRNA treatment relieved mechanical allodynia in IBD mice. Compared with the control + vectors group, the AWR score of CRD was increased in the TNBS + vectors group. The AWR score of CRD in the TNBS + P2Y12 shRNA group was lower than that in the TNBS + vectors group (Fig. 2c,  P < 0.05). The results indicated that P2Y12 shRNA treatment reversed visceral hypersensitivity in IBD mice.

To investigate whether P2Y12 shRNA treatment attenuated depression in IBD mice, SPT, TST and FST were used. Compared with the control + vectors group, the sucrose water intake was decreased in the TNBS + vectors group. The sucrose water intake in the TNBS + P2Y12 shRNA group was higher than that in the TNBS + vectors group (Fig. 2d,  P < 0.05). However, there was no significant difference in the proportion of floating time of each group in the FST (Fig. 2e, P  > 0.05). Compared with the control + vectors group, the proportion of immobility time was increased in the TNBS + vectors group. The proportion of immobility time in the TNBS + P2Y12 shRNA group was less than that in the TNBS + vectors group (Fig. 2f, P  < 0.05). The data demonstrated that P2Y12 shRNA relieved depression-like behaviors in IBD mice.

### P2Y12 shRNA treatment downregulated the expression of P2Y12 and IL-1β and weakened the activation of microglia in the mPFC of IBD mice

**Fig. 3:**
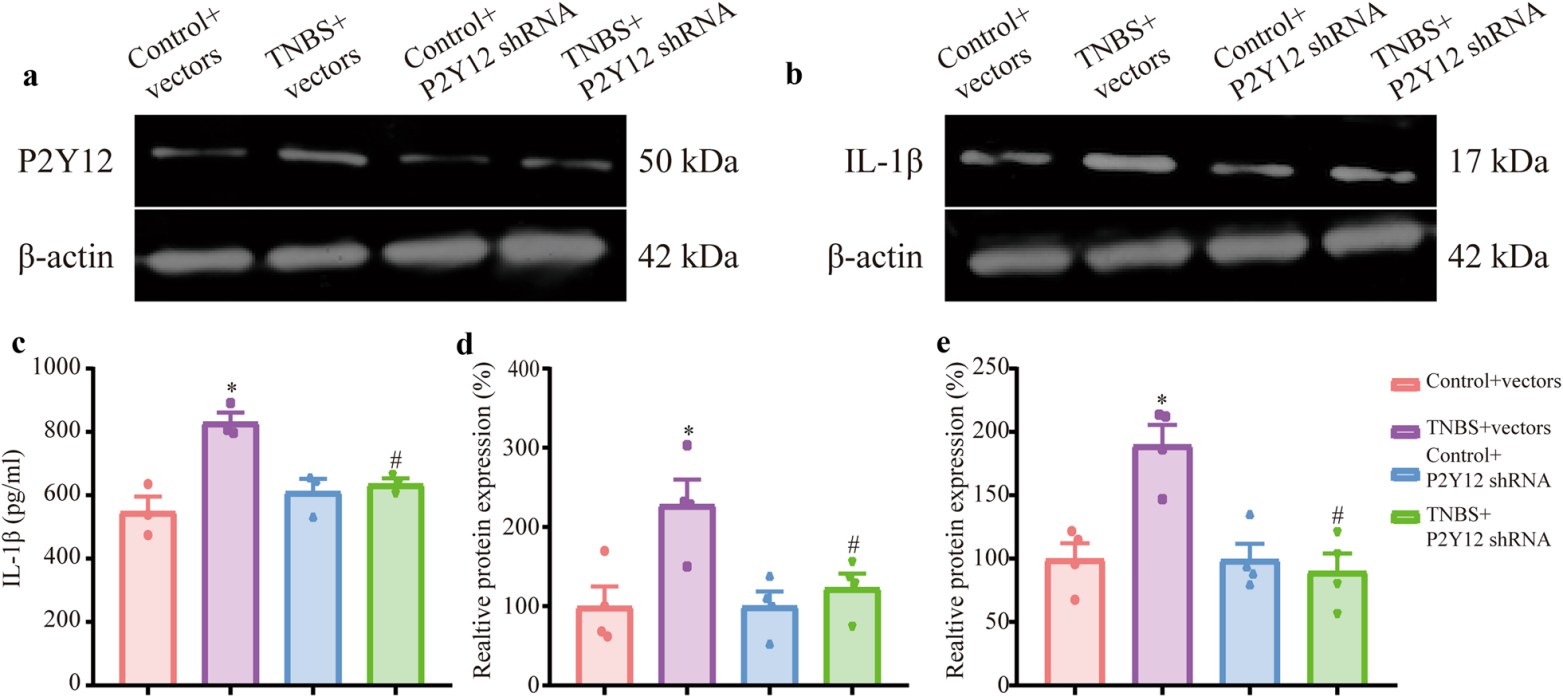
P2Y12 shRNA treatment downregulated the expression of P2Y12 and IL-1β in the mPFC. **a** Representative immunoblots of P2Y12 and β-actin protein expression in the mPFC. **b** Representative immunoblots of IL-1β and β-actin protein expression in the mPFC. **c** Enzyme-linked immunosorbent assay results. **d** Densitometric analysis of P2Y12 protein normalized to the loading control. **e** Densitometric analysis of IL-1β protein normalized to the loading control. Data are presented as the mean ± SEM (n = 3–4). *p < 0.05, compared with the control + vectors group; ^#^*p* < 0.05, compared with the TNBS + vectors group

To test whether injecting P2Y12 shRNA into the mPFC alters the expression of P2Y12 and IL-1β, western blotting and ELISA were used. Compared with that in the control + vectors group, the expression of P2Y12 was increased in TNBS + vectors group. Compared with TNBS + vectors group, the expression of P2Y12 was decreased in the TNBS + P2Y12 shRNA group (Fig. 3a, d, P  < 0.05). The expression of IL-1β in the TNBS + vectors group was higher than that in the control + vectors group. Compared with that in the TNBS + vectors group, the expression of IL-1β was decreased in the TNBS + P2Y12 shRNA group (Fig. 3b, e, P  < 0.05). ELISA showed similar trends of IL-1β by western blotting (Fig. 3c, P  < 0.05). In summary, the expression of IL-1β was downregulated by P2Y12 shRNA treatment in the mPFC of IBD mice.

**Fig. 4:**
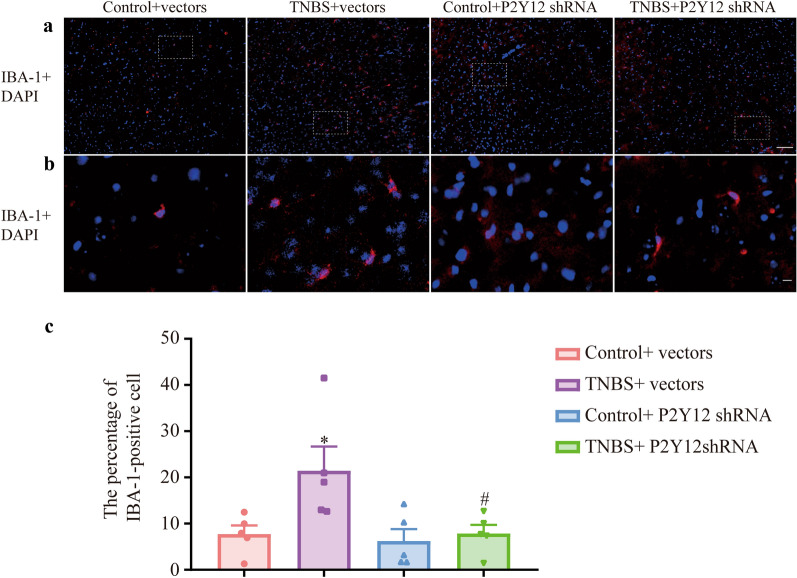
P2Y12 shRNA treatment changed the morphology of microglia in the mPFC of IBD mice. **a** IBA-1 are stained red and nuclei are stained blue with DAPI in mPFC of IBD mice. Scale bars: 200 μm. **b** IBA-1 are stained red and nucleus are stained blue with DAPI in the mPFC of IBD mice. Scale bars: 50 μm. **c** The percentage of IBA-1-positive cells, IBA-1-positive cells/somatic cells. Data are presented as the mean ± SEM (n = 5). *p < 0.05, compared with the control + vectors group; ^#^*p* < 0.05, compared with the TNBS + vectors group

**Fig. 5:**
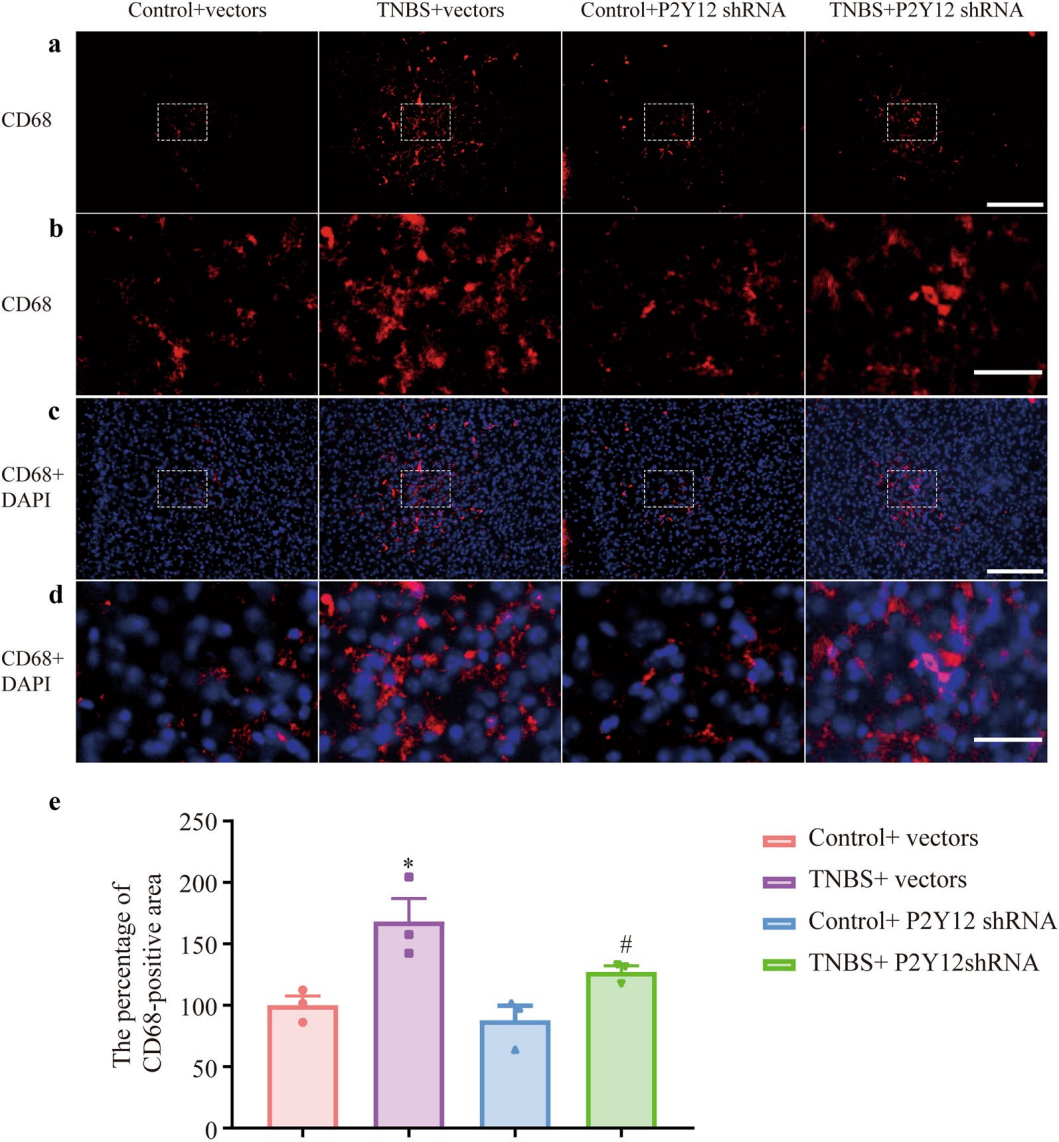
P2Y12 shRNA treatment inhibited the activation of microglia in the mPFC of IBD mice. **a** Representative images showing CD68 immunoreactivity in the mPFC of IBD mice. Represented microglial activated stained red with CD68. Scale bars: 200 μm. **b** Magnified representative images showing CD68 immunoreactivity in the mPFC of IBD mice. Represented microglial activated stained red with CD68. Scale bars: 50 μm. **c** CD68 are stained red and nuclei are stained blue with DAPI in the mPFC of IBD mice. Scale bars: 200 μm. **d** CD68 are stained red and nuclei are stained blue with DAPI in the mPFC of IBD mice. Scale bars: 50 μm. **e** The percentage of CD68-positive area. Data are presented as the mean ± SEM (n = 3). *p < 0.05, compared with the control + vectors group; ^#^*p* < 0.05, compared with the TNBS + vectors group

Microglia were stained red with IBA-1, and nuclei were stained blue with DAPI (Fig. 4a, b). Compared with the control + vectors group, the percentage of IBA-1-positive cells in the TNBS + vectors group was increased. Compared with that in the TNBS + vectors group, the percentage of IBA-1-positive cells in the TNBS + P2Y12 shRNA group was significantly decreased (Fig. 4c, P  < 0.05). Then, activated microglia were stained red with CD68 (Fig. 5a, b), and nuclei were stained blue with DAPI (Fig. 5c, d). Compared with the control + vectors group, the percentage of CD68-positive area in the TNBS + vectors group was increased. Compared with that in the TNBS + vectors group, the percentage of CD68-positive area in the TNBS + P2Y12 shRNA group was significantly decreased (Fig. 5e, P  < 0.05). The data demonstrated that microglial activation was inhibited by P2Y12 shRNA treatment in the mPFC of IBD mice.

### EA relieved the comorbidity of visceral pain and depression in IBD mice

**Fig. 6:**
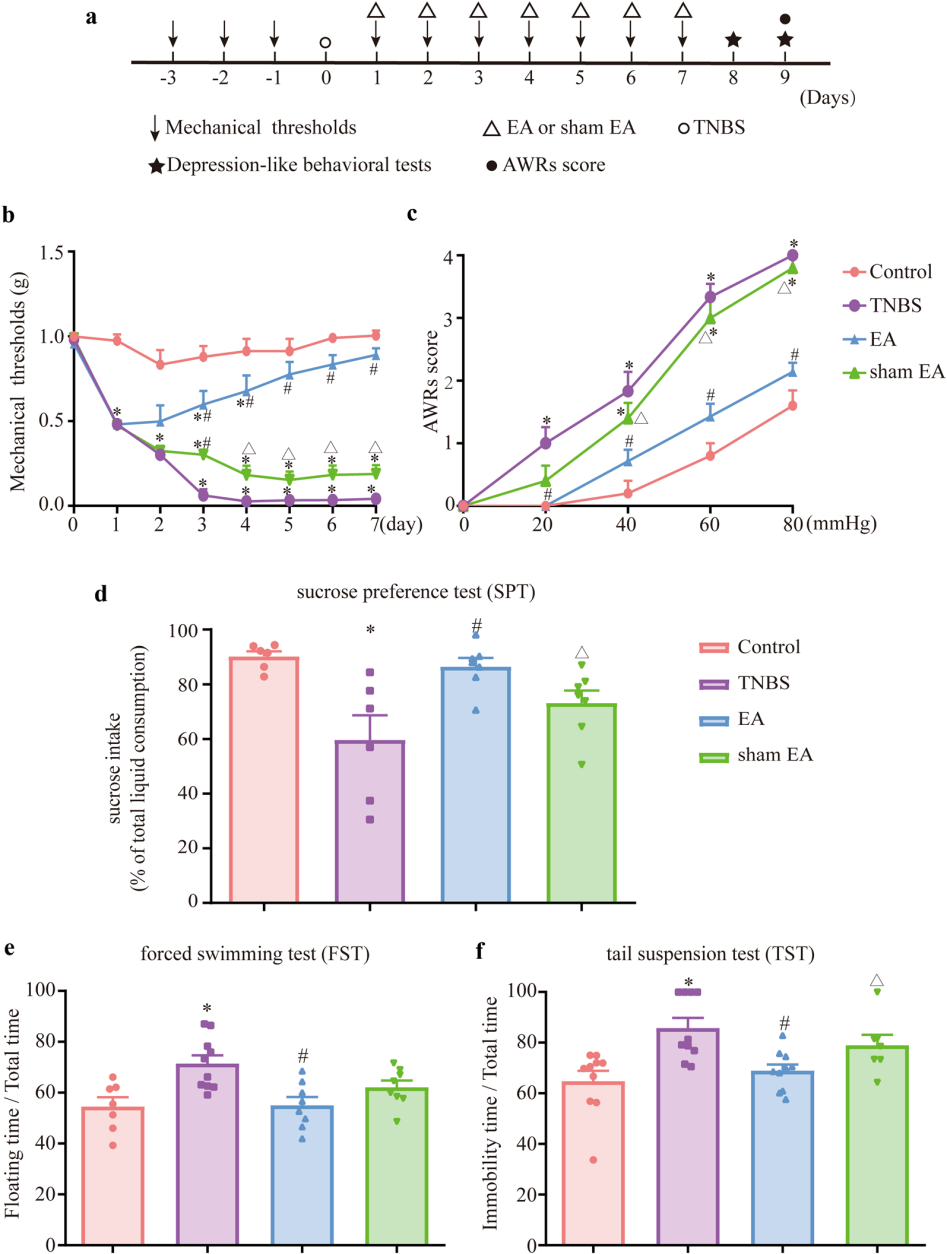
EA relieved mechanical allodynia, visceral hyperalgesia and depression-like behaviors in IBD mice. **a** Experimental flowchart. **b** Time course of mechanical threshold after EA treatment. **c** Visceral hyperalgesia was evaluated by CRD. **d** After EA treatment, mice’s preference for sucrose was recorded as: sucrose consumption(g)/(sucrose consumption(g) + water consumption(g)) × 100%. **e** Representative results of the effect of the EA treatment on the proportion of floating time in the FST. The proportion of floating time was recorded as: (Floating time(s) / total time(s)) × 100%. **f** Representative results of the effect of the EA treatment on the proportion of immobility time in the TST. The proportion of immobility time was recorded as: (Immobility time(s)/total time(g)) × 100%. The data are expressed as mean ± SEM (n = 7–10 mice). *p < 0.05, compared with the control group; ^#^*p* < 0.05, compared with the TNBS group; ^Δ^*p* < 0.05, compared with the EA group

Compared with the control group, the mechanical pain threshold was remarkably reduced in the TNBS group (Fig. 6b, P < 0.05), which indicated that TNBS caused mechanical allodynia in mice. On the 3rd day, compared with the TNBS group, the mechanical pain threshold was significantly increased in the EA but not the sham EA group (Fig. 6b, P  < 0.05). On the 4th day, compared with the sham EA group, the mechanical pain threshold was significantly increased in the EA group (Fig. 6b, P  < 0.05). The data indicated that EA relieved mechanical allodynia in IBD mice and that sham EA was not effective. The AWR score of CRD in the TNBS group was higher than that in the control group (Fig. 6c, P  < 0.05). Compared with the TNBS group, the AWR score of CRD in the EA but not the sham EA group significantly decreased (Fig. 6c, P  < 0.05). Compared with the sham EA group, the AWR score of the CRD was significantly decreased in the EA group (Fig. 6c, P  < 0.05). The results indicated that EA reversed visceral hypersensitivity in IBD mice.

In the SPT, compared with the control group, the sucrose water intake in the TNBS group was decreased (Fig. 6d, P  < 0.05). Compared with the TNBS group, the sucrose water intake in the EA but not the sham EA group was increased (Fig. 6d, P  < 0.05). Compared with the sham EA group, the sucrose water intake in the EA group was increased (Fig. 6d, P  < 0.05). In the TST and FST, compared with the control group, the proportion of floating time and the proportion of immobility time in the TNBS group were increased (Fig. 6e, f, P  < 0.05). Compared with the TNBS group, the proportion of floating time and the proportion of immobility time in the EA but not sham the EA group were decreased (Fig. 6e, f, P  < 0.05). There was no difference between the EA group and the sham EA group in the FST (Fig. 6e, P  < 0.05). In the TST, compared with the sham EA group, the proportion of immobility time in the EA group was decreased (Fig. 6f, P  < 0.05). Taken together, EA relieved depression-like behaviors in IBD mice.

### EA downregulated the expression of P2Y12 and IL-1β and weakened abnormal activation of microglia in the mPFC of IBD mice

**Fig. 7:**
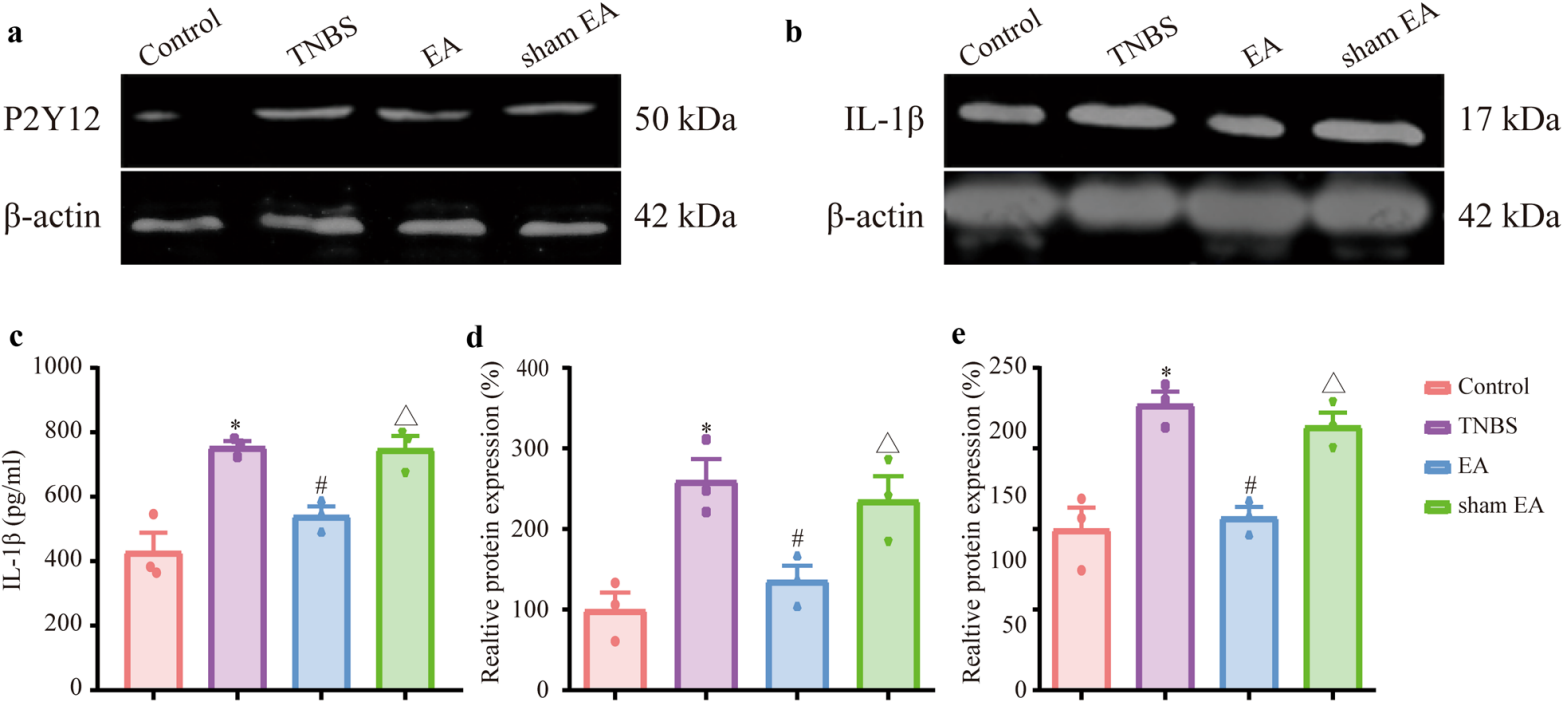
EA downregulated the expression of P2Y12 and IL-1β in the mPFC. **a** Representative immunoblots of P2Y12 and β-actin protein expression in the mPFC. **b** Representative immunoblots of IL-1β and β-actin protein expression in the mPFC. **c** Enzyme-linked immunosorbent assay results. **d** Densitometric analysis of P2Y12 protein normalized to the loading control. **e** Densitometric analysis of IL-1β protein normalized to the loading control. Data are presented as the mean ± SEM (n = 3). *p < 0.05, compared with the control group; ^#^*p* < 0.05, compared with the TNBS group; ^Δ^*p* < 0.05, compared with the EA group

The expression of P2Y12 in the TNBS group was higher than that in the control group (Fig. 7a, d, P  < 0.05). EA but not sham EA downregulated the expression of P2Y12 compared with the TNBS group (Fig. 7a, d, P  < 0.05). Compared with that in the sham EA group, the expression of P2Y12 in the EA group was downregulated (Fig. 7a, d, P  < 0.05). Compared with the control group, the expression of IL-1β was increased in the TNBS group (Fig. 7b, e, P  < 0.05). Compared with that in the TNBS group, the expression of IL-1β in the EA group but not the sham EA group was downregulated (Fig. 7b, e, P  < 0.05). Compared with that in the sham EA group, the expression of IL-1β in the EA group was downregulated (Fig. 7b, e, P  < 0.05). ELISA showed similar trends of IL-1β by western blotting (Fig. 7c, P  < 0.05). In summary, EA downregulated the expression of P2Y12 and inhibited IL-1β expression in the mPFC of IBD mice.

**Fig. 8:**
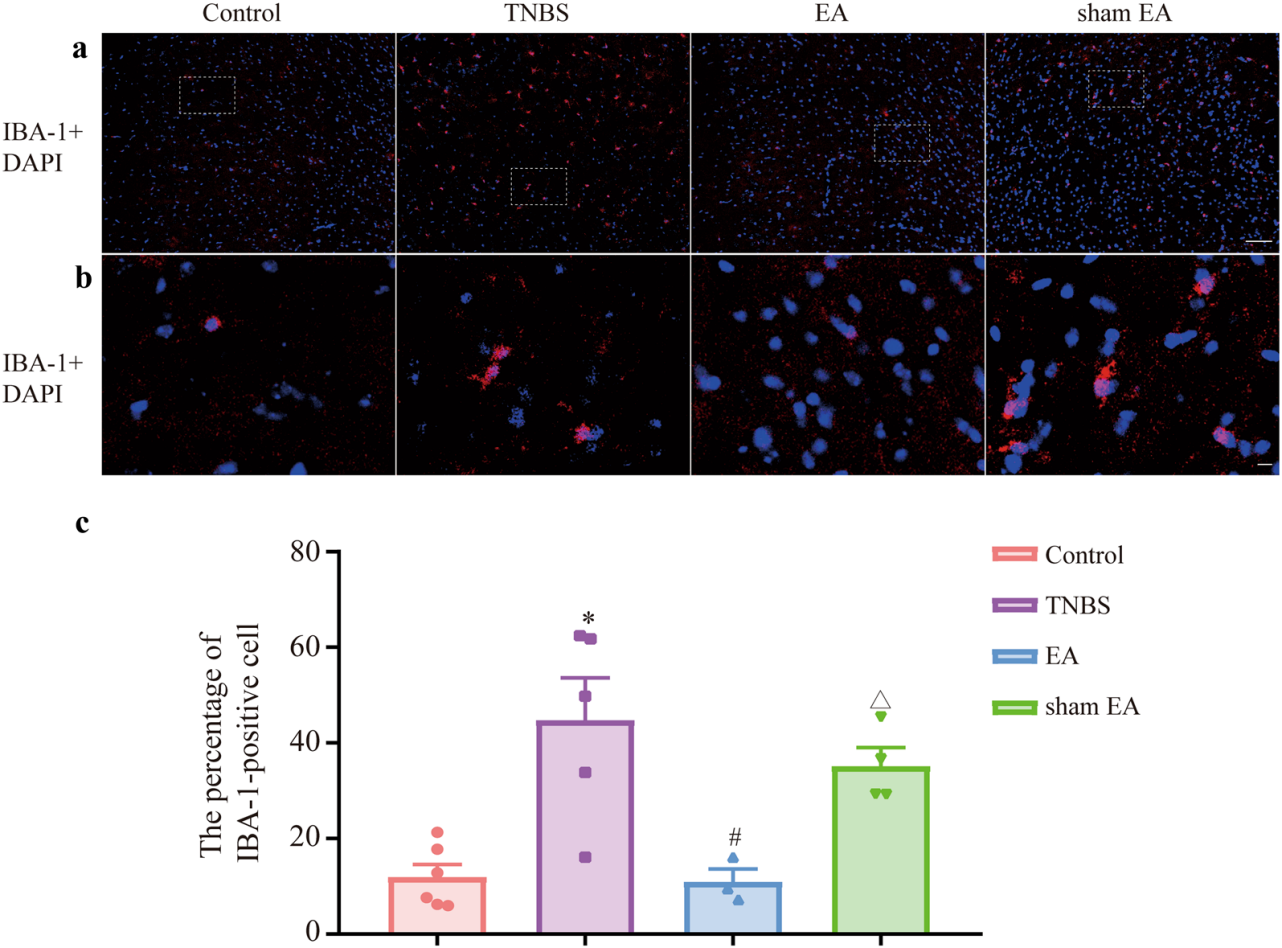
EA changed the morphology of microglia in the mPFC of IBD mice. **a** IBA-1 are stained red and nuclei are stained blue with DAPI in the mPFC of IBD mice. Scale bars: 200 μm. **b** IBA-1 are stained red and nuclei are stained blue with DAPI in the mPFC of IBD mice. Scale bars: 50 μm. **c** The percentage of IBA-1-positive cells, IBA-1-positive cells/somatic cells. Data are presented as the mean ± SEM (n = 3–6). *p < 0.05, compared with the control + vectors group; ^#^*p* < 0.05, compared with the TNBS + vectors group; ^Δ^*p* < 0.05, compared with the EA group

**Fig. 9:**
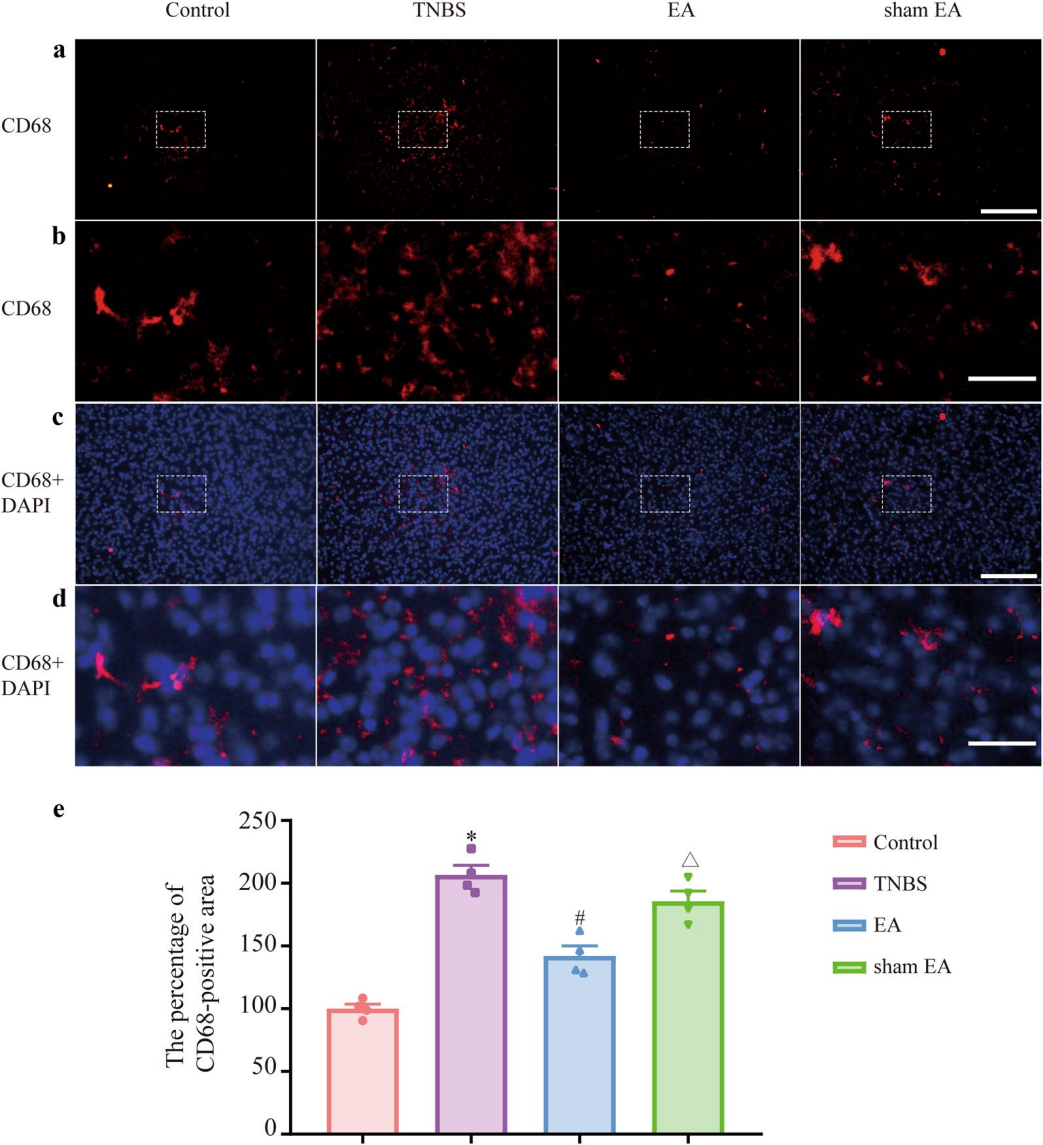
EA inhibited the activation of microglia in the mPFC of IBD mice. **a** Representative images showing CD68 immunoreactivity in the mPFC of IBD mice. Represented microglial activated stained red with CD68. Scale bars: 200 μm. **b** Magnified representative images showing CD68 immunoreactivity in the mPFC of IBD mice. Represented microglial activated stained red with CD68. Scale bars: 50 μm. **c** CD68 are stained red and nuclei are stained blue with DAPI in the mPFC of IBD mice. Scale bars: 200 μm.** d** CD68 are stained red and nuclei are stained blue with DAPI in the mPFC of IBD mice. Scale bars: 50 μm. **e** The percentage of CD68-positive area. Data are presented as the mean ± SEM (n = 4). *p < 0.05, compared with the control group; ^#^*p* < 0.05, compared with the TNBS group; ^Δ^*p* < 0.05, compared with the EA group

Microglia were stained red with IBA-1, and nuclei were stained blue with DAPI (Fig. 8a, b). The percentage of IBA-1-positive cells in the TNBS group was higher than that in the control group. EA but not sham EA decreased the percentage of IBA-1-positive cells compared with the TNBS group. Compared with that in the sham EA group, the percentage of IBA-1-positive cells in the EA group was downregulated (Fig. 8c, P  < 0.05). Then, activated microglia were stained red with CD68 (Fig. 9a, b), and nuclei were stained blue with DAPI (Fig. 9c, d). Compared with the control group, the percentage of CD68-positive area in the TNBS group was increased. Compared with that in the TNBS group, the percentage of CD68-positive area in the EA group was significantly decreased (Fig. 9e, P  < 0.05). Compared with that in the sham EA group, the percentage of CD68-positive area in the EA group was decreased (Fig. 9e, P < 0.05). The data demonstrated that microglial activation was inhibited by EA in the mPFC of IBD mice.

## Discussion

IBD is defined as chronic relapsing bowel inflammation, including Crohn's disease and ulcerative colitis [28]. As the incidence and prevalence of IBD is increasing worldwide, it has become a shared health care problem [29]. Visceral pain is one of the main symptoms of IBD and seriously reduces quality of life [30]. In addition to causing inflammatory reactions, such as visceral pain, IBD is often accompanied by emotional symptoms, such as anxiety and depression [31, 32]. In the past few decades, physicians have focused on the treatment of gastrointestinal symptoms, visceral abdominal pain and diarrhea of IBD but have ignored the accompanying anxiety, depression and other emotional symptoms. In this study, we found that comorbidity of visceral pain and depression-like behaviors occurred in IBD mice and that the expression of P2Y12 was increased in the mPFC of IBD mice. Meanwhile, P2Y12 shRNA alleviated mechanical pain allodynia and visceral hyperalgesia in IBD mice and improved their depression-like behaviors. EA inhibited the expression of P2Y12 and IL-1β in the mPFC and weakened the activation of microglia, thus relieving visceral pain and depression in IBD mice. Therefore, our study provides new information that the P2Y12 receptor is a new target for the clinical treatment of comorbid visceral pain and depression in IBD, which improves our understanding of the underlying mechanisms of EA.

Purinergic receptors play an important role in neurotransmission and gastrointestinal secretory motor functions [33]. The P2Y12 receptor is a metabotropic G protein-coupled purinergic receptor that was first found in platelets and later found to exist in microglia of the central nervous system (CNS) [34–37]. Studies have shown that the P2Y12 receptor is closely related to cancer pain [9] and neuropathic pain [38]. The mPFC is important not only in pain processing but also in the onset of depression-like behaviors [39, 40]. In this study, we found that the expression of P2Y12 was upregulated in the mPFC of IBD mice. Interference with P2Y12 relieved the comorbidity of visceral pain and depression in IBD mice. These results suggest that the P2Y12 receptor in the mPFC could be a new target for the clinical treatment of comorbid visceral pain and depression.

Previous studies have revealed that the expression of the inflammatory factors IL-1β, interleukin-6 (IL-6) and tumor necrosis factor-α (TNF-α) in the spinal cord is inhibited by selective antagonists of the P2Y12 receptor, which relieves acute and chronic inflammatory pain [9]. In clinical and basic experiments, lines of evidence demonstrated that inflammatory factors not only mediate visceral hyperalgesia but also play a vital role in the comorbidity of visceral pain and depression [41, 42]. Among them, IL-1β is the key molecule mediating the depression-like behaviors induced by acute and chronic stress [12]. For the first time, this study showed that the protein expression of P2Y12 and IL-1β was significantly increased in IBD mice. Interference with the expression of P2Y12 in the mPFC reduced the expression of IL-1β, suggesting that P2Y12 may be upstream of IL-1β. Thus, downregulation of P2Y12 in the mPFC decreased the expression of IL-1β and alleviated the comorbidity of visceral pain and depression in IBD mice. However, this study lacks research on IL-6 and TNF-α, and the mechanism is not deeply explored.

Microglial cells are the primary immune cells of the CNS and play an important role in brain homeostasis and neurological diseases [43]. Previous studies have shown that the activation of the P2Y12 receptor is mainly related to the damage or pathological state of microglia, which in turn activates microglia and causes them to secrete IL-1β [44]. Blocking P2Y12 receptors may inhibit microglial activation, prevent the formation of platelet aggregates, and reduce proinflammatory cytokine levels, which means that P2Y12 inhibitors have not only antithrombotic effects but also neuroprotective effects [45]. IBA-1 is a marker of microglia and CD68 is a marker of activated microglia [46]. Our results showed that both the percentage of IBA-1-positive cells and the percentage of CD68-positive area was significantly increased in IBD mice, indicating that microglia were significantly activated. After P2Y12 shRNA treatment, the activated microglia in the mPFC was decreased. Inhibiting the expression of P2Y12 in the mPFC alleviated visceral pain and depression in IBD mice, weakened the activation of microglia, and reduced the expression of IL-1β in the mPFC. This may be the reason why P2Y12 shRNA treatment alleviated the comorbidity of visceral pain and depression in IBD mice.

**Fig. 10:**
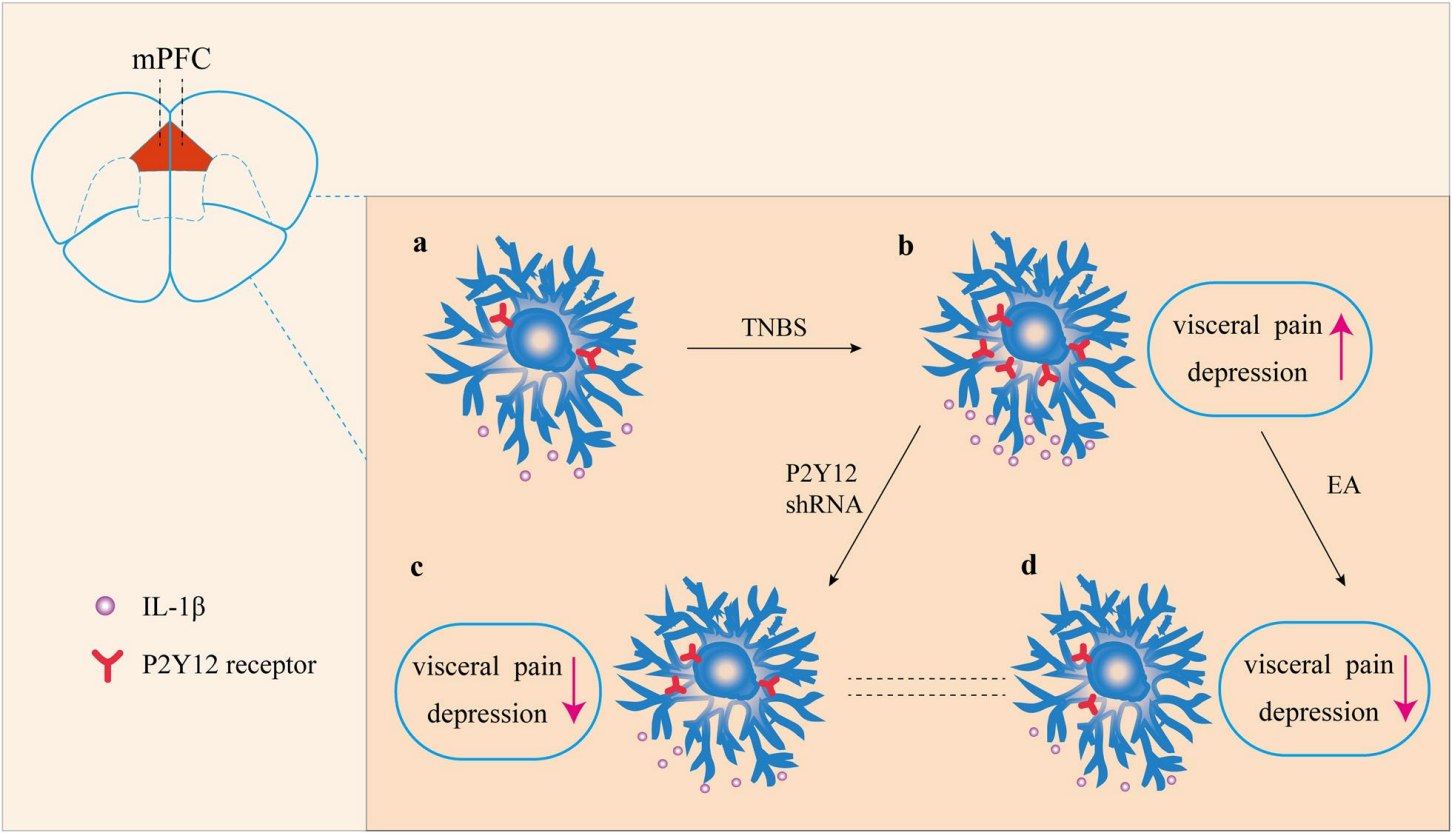
P2Y12 receptor play an important role in IBD mice. **a** The expression of P2Y12 and IL-1β in mPFC of C57 mice. **b** Comorbidity of visceral pain and depression-like behaviors occurred in IBD mice and the expression of P2Y12 and IL-1β is increased in mPFC of IBD mice. **c** P2Y12 shRNA down-regulated the expression of P2Y12 and inhibited IL-1β expression in mPFC, thus relieving visceral pain and depression of IBD mice. **d** EA down-regulated the expression of P2Y12 and inhibited IL-1β expression in mPFC, thus relieving visceral pain and depression of IBD mice

As a part of complementary and alternative medicine (CAM), EA has proven to be effective in the treatment of chronic pain [47]. Previous research proved that EA has a significant analgesic effect on visceral pain in mice with IBD [26]. However, it urgently needs to be explored whether EA can improve the incidence of comorbidity of visceral pain and depression. In this study, we found that IBD mice showed obvious mechanical allodynia and visceral hyperalgesia, which were significantly inhibited by EA at acupoint BL25 at frequencies of 1 mA and 2 Hz. These results were consistent with previous research [26]. Interestingly, EA also alleviated depression-like behaviors. For the first time, our results demonstrated the antidepressant effect of EA on IBD mice. EA decreased the expression of P2Y12 and IL-1β as well as the activation of microglia, suggesting that EA downregulated the expression of P2Y12, weakened the activation of microglia, and then inhibited IL-1β expression in the mPFC, thus relieving hyperalgesia and depression in IBD mice (Fig. 10).

## Conclusion

The P2Y12 receptor is a purinoceptor that is engaged in platelet aggregation, and P2Y12 inhibitors have been widely used in clinical antithrombotic therapy. For the first time, we found that upregulation of the P2Y12 receptor in the mPFC is associated with the comorbidity of visceral pain and depression. Interference with both P2Y12 shRNA and EA relieved the comorbidity of visceral pain and depression in IBD mice. P2Y12 shRNA and EA significantly downregulated the expression of P2Y12, weakened the activation of microglia, and then inhibited IL-1β expression in the mPFC, thus relieving visceral pain and depression-like behaviors in IBD mice. The present study provided new ideas suggesting that the P2Y12 receptor in the mPFC could be a new target for the clinical treatment of the comorbid visceral pain and depression by EA. This may not only deepen our understanding of the analgesic and antidepressant mechanisms of EA but also promote the application of EA in the clinical treatment of IBD-induced comorbid visceral pain and depression.

## Supplementary Information

**Additional file 1: Fig. S1. a** The picture of injection site. **b** The picture of infection area. **c** The P2Y12 level of mRNA in mPFC.

## Data Availability

Not applicable.

## Abbreviations

IL-1β: Induce interleukin-1β
EA: Electroacupuncture
IBD: Inflammatory bowel disease
mPFC: Medial prefrontal cortex
TNBS: 2,4,6-Trinitrobenzene sulfonic acid
CRD: Colorectal distension
SPT: Sucrose preference test
TST: Tail suspension test
FST: Forced swimming test
shRNA: Short hairpin RNA
AWRs: Abdominal withdrawal reflex
CNS: Central nervous system
IL-6: Interleukin-6
TNF-α: Tumor necrosis factor-α
CAM: Complementary and alternative medicine
