# Supplementary figures and images for: P2Y12 receptor as a new target for electroacupuncture relieving comorbidity of visceral pain and depression of inflammatory bowel disease
Source: Chin Med. 2021 Dec 20;16:139. doi: 10.1186/s13020-021-00553-9 (PMC8686637; doi:10.1186/s13020-021-00553-9)

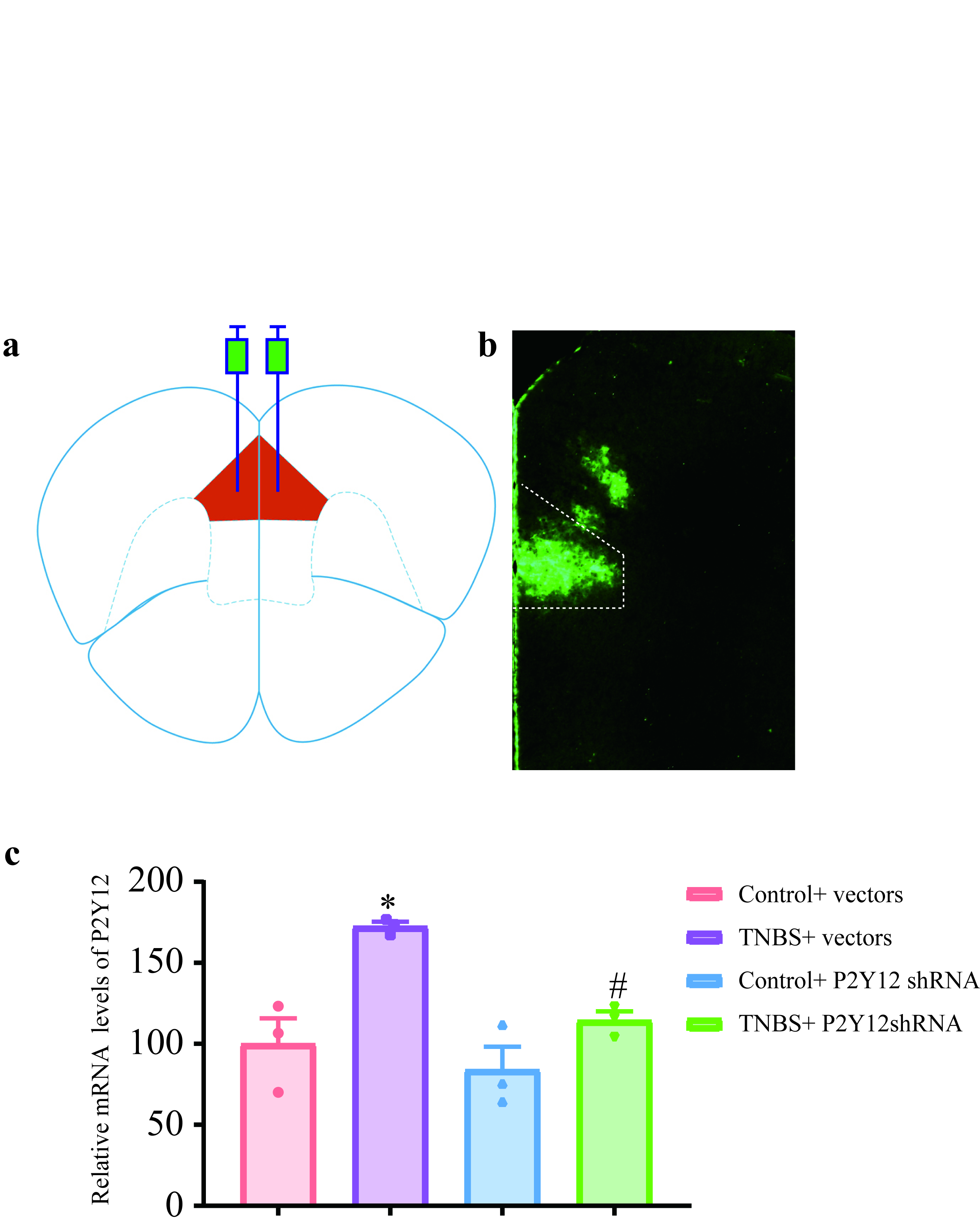

Supplement: Supplementary file 1 — Additional file 1: Fig. S1. a The picture of injection site. b The picture of infection area. c The P2Y12 level of mRNA in mPFC. [file 13020_2021_553_MOESM1_ESM.tif]
